# Cross-reactivity of antibodies against interferon beta in multiple sclerosis patients and interference of the JAK-STAT signaling pathway

**DOI:** 10.1038/s41598-017-16828-x

**Published:** 2017-11-29

**Authors:** Isaac Hurtado-Guerrero, Maria Jesus Pinto-Medel, Patricia Urbaneja, Jose Luis Rodriguez-Bada, Jesús Ortega-Pinazo, Pedro Serrano, Óscar Fernández, Laura Leyva, Begoña Oliver-Martos

**Affiliations:** 1 Unidad de Gestión Clínica de Neurociencias, Instituto de Biomedicina de Málaga (IBIMA), Hospital Regional Universitario de Málaga, Universidad de Málaga, Málaga, Spain; 2 Red Española de Esclerosis Multiple (REEM), Instituto de Salud Carlos III, Madrid, Spain

## Abstract

Interferon beta (IFNβ) therapy has immunogenic properties and induces the development of neutralizing antibodies (NAbs). From the extensive literature focused in the development of NAbs in multiple sclerosis (MS) patients, their ability to cross-react has been deficiently evaluated, despite having important consequences in the clinical practice. Here, the relation between the cross-reactivity and the NAbs titers has been evaluated in MS patients, by inhibition of the antiviral activity of IFNβ by bioassay and through the interference with the activation of the IFNß pathway (JAK-STAT), by phosphoflow. Thus, patients with intermediate-high titers of NAbs, determined by bioassay, had a 79-fold increased risk of cross-reactivity compared to patients with low titers. The cross-reactivity is also demonstrated because NAbs positive sera were able to decrease significantly the activation of pSTAT1 achieved by other different IFNβ molecules in the cells patients. Besides, a linear relationship between the STAT1 phosphorylation and NAbs titers was found. The study demonstrates that cross-reactivity increases with the titer of antibodies, which has important implications in clinical practice when switching the treatment. The direct relationship between the NAbs titer and the activation of STAT1 suggest that its determination could be an indirect method to identify the presence of NAbs.

## Introduction

During last years, several drugs have been approved for the treatment of multiple sclerosis (MS), nonetheless, interferon beta (IFNβ) continues being a widely used treatment for newly diagnosed patients with relapsing-onset multiple sclerosis. Like other therapeutic proteins, IFNβ has immunogenic potential when administered therapeutically. A variable number of patients, between 2% and 45%^1^, develop neutralizing antibodies (NAbs) that bind to specific residues of the IFNβ molecule, blocking the interaction with its cell surface receptor. There has been much controversy about the significance of these antibodies in IFNβ treated patients and its use in clinical practice, but it is widely accepted that high and permanent titers of NAbs have to be taken into consideration since they abolishes IFNβ activity and could be related with treatment inefficacy^2^.

Exogenous IFNβ is a recombinant protein available in three formulations, two as IFNβ-1a (intramuscular (i.m.) and subcutaneous (s.c.)) which are produced in mammalian cells with identical structure to the native form, and one as IFNβ-1b (s.c.), that is produced in Escherichia coli and differs from native form, mainly by lack of glycosylation. These differences in the biochemical structure, as well as in the excipients, route and timing of administration, translate into different immunogenicity among preparations. Moreover, the differences regarding the definition of NAbs positivity, the tests used to assess the NAbs, and the design of the studies result in a wide interval of positive patients described^3^.

In spite of the differences between IFNβ-1a and IFNβ-1b, neutralizing antibodies generated against one of the IFNβ preparations are able to bind and neutralize other different IFNβ preparations that have not induced their formation, a fact known as cross-reactivity. The ability of NAbs to cross-react has important implications from a clinical point of view when having to take a decision about switching therapies and compromise further therapy. But also, the antibodies against therapeutic proteins can have serious consequences for the patient because they are able to cross react and neutralize the native proteins, with the consequent induction of adverse events. This fact has been described for thromboepoetin (TPO), which induces the formation of antibodies that neutralize native TPO and cause thrombocytopenia^4^ but also with IFNβ, whose antibodies neutralize endogenous IFNβ^5^.

Notwithstanding the aforementioned issues, from the extensive literature focused in the study of NAbs in MS patients, few studies and with limited number of patients have been conducted with regard to cross-reactivity^6–10^.

Our expertise as center for the determination of NAbs has allowed us to evaluate its cross-reactivity in an important cohort of MS patients, not only by inhibition of the antiviral activity of IFNβ by bioassay but also through evaluation of the interference with the JAK-STAT signaling pathway, which is the pathway through which IFNß exerts its function, by phosphoflow cytometry. In addition, the relationship between the antibody titer and the activation by phosphorylation of STAT1 has been assessed.

## Results

### Relation between cross-reactivity and neutralizing antibody titers

According to the established categories of NAbs titers, 63 patients (28.4%) had low (≤100 TRU/ml), 27 (12.2%) had intermediate (>100 and ≤300 TRU/ml) and 132 (59.4%) had high titers (>300 TRU).

Subsequently, the percentage of patients who presented cross-reactivity according to the antibody titers was calculated. Thus, the cross-reactivity was observed in 42 out of 63 patients with low (66.7%), in all the patients with intermediate (100%) and in 131 out of 132 (99.2%) patients with high titers of NAbs (Table 1).

**Table 1:** Neutralizing antibody titers and cross-reactivity in MS patients treated with IFNβ.

Neutralizing antibody		Patients with cross-reactivity	Patients without cross-reactivity
Low (≤100 TRU/ml)	63 (28.4%)	42 (66.7%)	21 (33.3%)
Intermediate (>100 and ≤300 TRU/ml)	27 (12.2%)	27 (100%)	—
High (>300 TRU/mL)	132 (59.4%)	131 (99.2%)	1 (0.8%)

Statistical analysis using chi-square test showed a relationship between the antibody titers and the presence of cross-reactivity (p < 0.0001), so that the number of individuals with cross-reactivity increases as higher the antibody titer is, presenting cross reactivity almost always in those individuals with NAbs greater than 100 TRU/ml.

Then, patients with intermediate and high titers were grouped and a logistic regression analysis was performed to calculate the risk of cross-reactivity according to NAbs titers. The first model shows that patients with intermediate-high titers have a significant risk of 79-fold of cross-reactivity compared to patients with low titers (p = 2,5e-005) (Table 2).

**Table 2:** Logistic regression analysis of cross-reactivity risk according to the antibody titter.

Logistic regression models	NAbs titer	OR	IC 95%	P_value_
**Model 1** Risk of cross-reactivity	Intermediate-high *vs* Low	79	10.33–604.37	2.5 × 10^−5^
**Model 2** Risk of cross-reactivity with one molecule	Intermediate-high *vs* Low	36	4.27–308.02	0.001
**Model 3** Risk of cross-reactivity with two molecule	Intermediate-high *vs* Low	124	15.85–983.96	4.5 × 10^−6^

OR: Odd ratio; IC: confidence interval; p: p values.

After that, a second model to calculate the risk of cross-reactivity with one or two of the IFNβ molecules according to NAbs titers was constructed. Patients with intermediate-high titers have a significant risk of 36-fold to cross react with only one of the IFNβ molecule different from the immunogen that patients with low titers (p = 0.001), and a significant risk of 124-fold to cross react with two IFNβ molecules different from the immunogen (p = 4, 5e-006) (Table 2).

Finally, in the ROC curves analysis of the predicted probability for the first model, an area under the curve of 0.872 (CI: 0.809–0.936) was obtained and was statistically significant (p = 1.01e-008).

To evaluate the risk of cross-reactivity with only one of the IFNβ molecules according to NAbs titers, with the second model, an area under the curve of 0.794 (CI: 0.670–0.918; p = 0.0003) was obtained. The predictive capacity was increased to evaluate risk of cross-reactivity with the two IFNβ molecules, with an area under the curve of 0.905 (CI: 0.843–0.968; p = 1,22e-009).

### Interference of the presence of neutralizing antibodies in the activation of the IFNβ signaling pathway and cross-reactivity

NAbs circulating in the patient’s serum were generated against the molecule of IFNβ which was receiving as therapy, but as demonstrated by bioassay, they were able to block the antiviral activity of the other IFNβ molecules. In this *in vitro* study, it has been evaluated: 1. whether the presence of NAbs decreases the activation of pSTAT1 in reference to the activation achieved without NAbs and 2. whether these antibodies are able to decrease the activation of pSTAT1 achieved with another commercial IFNβ molecules (cross-reactivity). Consequently, PBMC from NAbs positive patients were cultured under stimulation with IFNβ and in the presence of NAbs positive autologous serum and B18R inhibitor.

**Figure 1:**
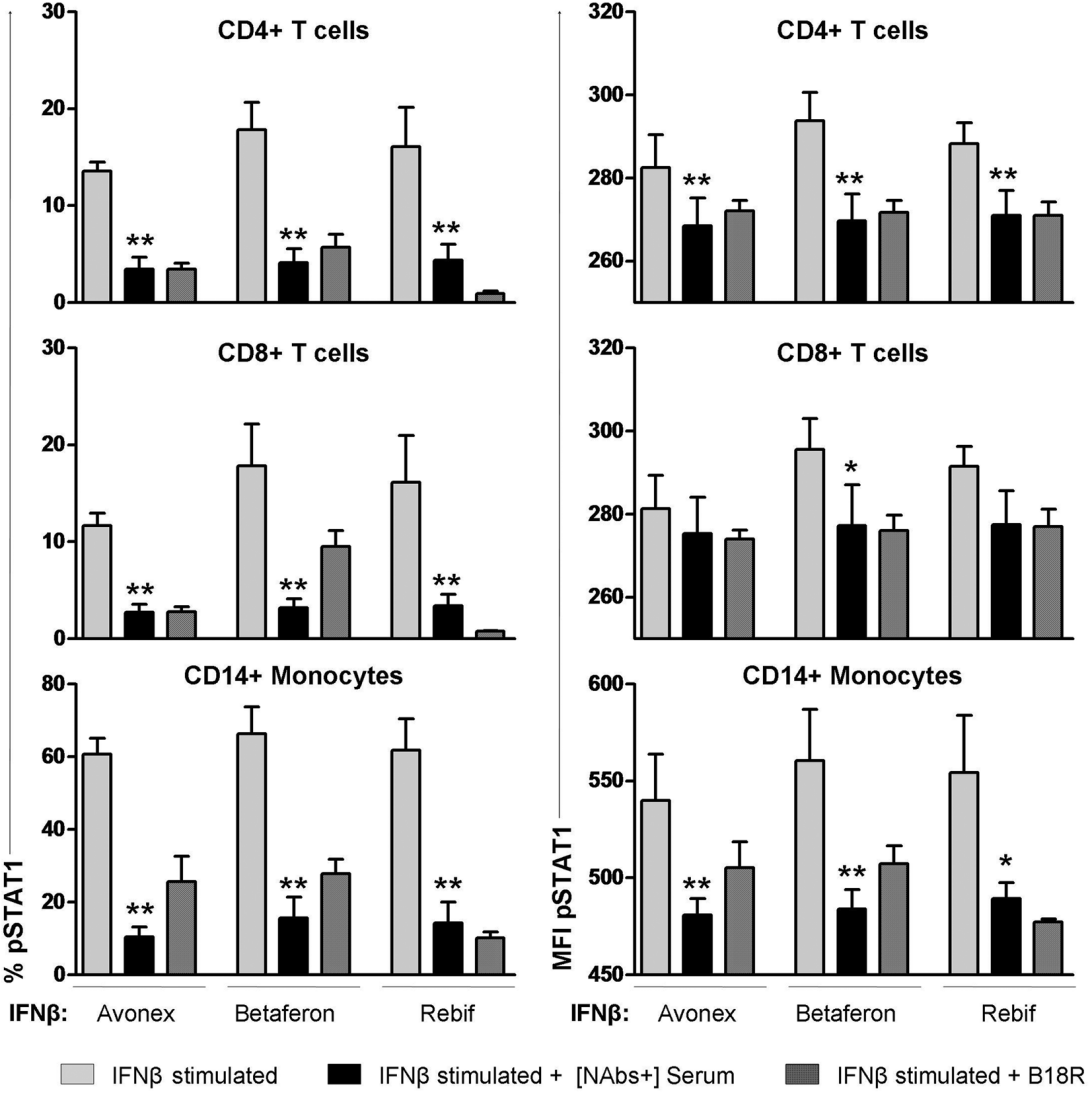
Interference of the presence of NAbs positive autologous serum in STAT1 activation and cross-reactivity. Expression of pSTAT1 quantified by phosphoflow in monocytes, CD4+ and CD8+ T lymphocytes from MS patients after stimulation with 1000 IU/mL IFNβ (Avonex, Betaferon or Rebif), in presence of autologous serum with NAbs generated against IFNβ1a (Rebif) received as treatment, and in presence of B18R inhibitor. The activation of pSTAT1 reached after IFNß stimulation decreased significantly in presence of NAbs positive autologous serum compared to the activation without serum in T cells and monocytes. NAbs generated against Rebif, were able to cross-react and to decrease significantly pSTAT1 expression achieved with the other two IFNβ molecules in the three subpopulation analyzed. Data are shown as MFI and percentage of cells expressing pSTAT1. Significant p values are shown with asterisk, *p ≤ 0.05; **p ≤ 0.01.

The activation of the JAk-STAT pathway after stimulation with Betaferon, Avonex and Rebif, determined as the % of cells expressing pSTAT1, decreased significantly in the presence of autologous NAbs positive serum compared to the activation reached in the absence of serum, in T cells (CD4+ and CD8+) and monocytes (Fig. 1). Similarly, MFI decreased significantly in CD4+ and monocytes when antibodies were present. This decrease was also observed in CD8+ T cells although it did not reach significance. When the inhibitor B18R was present, the expression of pSTAT1 as % of cells and MFI decreased in the three subpopulations analyzed.

Regarding the cross-reactivity, all the antibodies were generated against Rebif, which was the molecule received as therapy, and these NAbs were able to decrease significantly the MFI and the % of cells expressing pSTAT1 achieved with stimulation with the other two IFNβ molecules in the three subpopulations analyzed (Fig. 1).

Moreover, the percentage of inhibition in presence of NAbs (respect to the activation achieved with each IFNβ molecule) was calculated to evaluate if antibodies generated against Rebif inhibited equally the activation achieved with Betaferon and Avonex. There were no significant differences between the inhibition of pSTAT1 MFI of each molecule [Avonex CD4+ (75.74 ± 25.50), Betaferon CD4+ (78.89 ± 19.89), Rebif CD4+ (76.92 ± 11.85)] (graphic not shown).

### Inhibition of STAT1 phosphorylation according to the NAbs titers

First, we demonstrated that the decrease in the percentage of pSTAT1+ cells occurred not only in the presence of an autologous NAbs positive sera but also in the presence of an hererologous NAb positive sera. However, an autologous or heterologous NAbs negative sera never decreased the levels of pSTAT1+ cells, as shown in Supplementary Figure 1.

Then, the STAT1 phosphorylation was determined after IFNβ stimulation in the presence of serial dilutions of a pool of heterologous NAbs positive sera. As controls, unstimulated cells, IFNβ stimulated cells with the B18R inhibitor, and IFNβ stimulated cells with a pool of heterologous NAbs negative sera, were included.

As expected with the controls, the STAT1 phosphorylation obtained in IFNβ stimulated cells was significantly higher compared to those obtained in non stimulated cells in CD4+, CD8+ T cells and monocytes (p = 0.012 for the 3 subsets). However, the STAT1 phosphorylation obtained in IFNβ stimulated cells in the presence of B18R was significantly lower than the phosphorylation achieved in IFNβ stimulated cells, in CD4+, CD8+ T cells and monocytes (p = 0.012, p = 0.012 and p = 0.025 respectively) and similar to those obtained in non stimulated cells.

**Figure 2:**
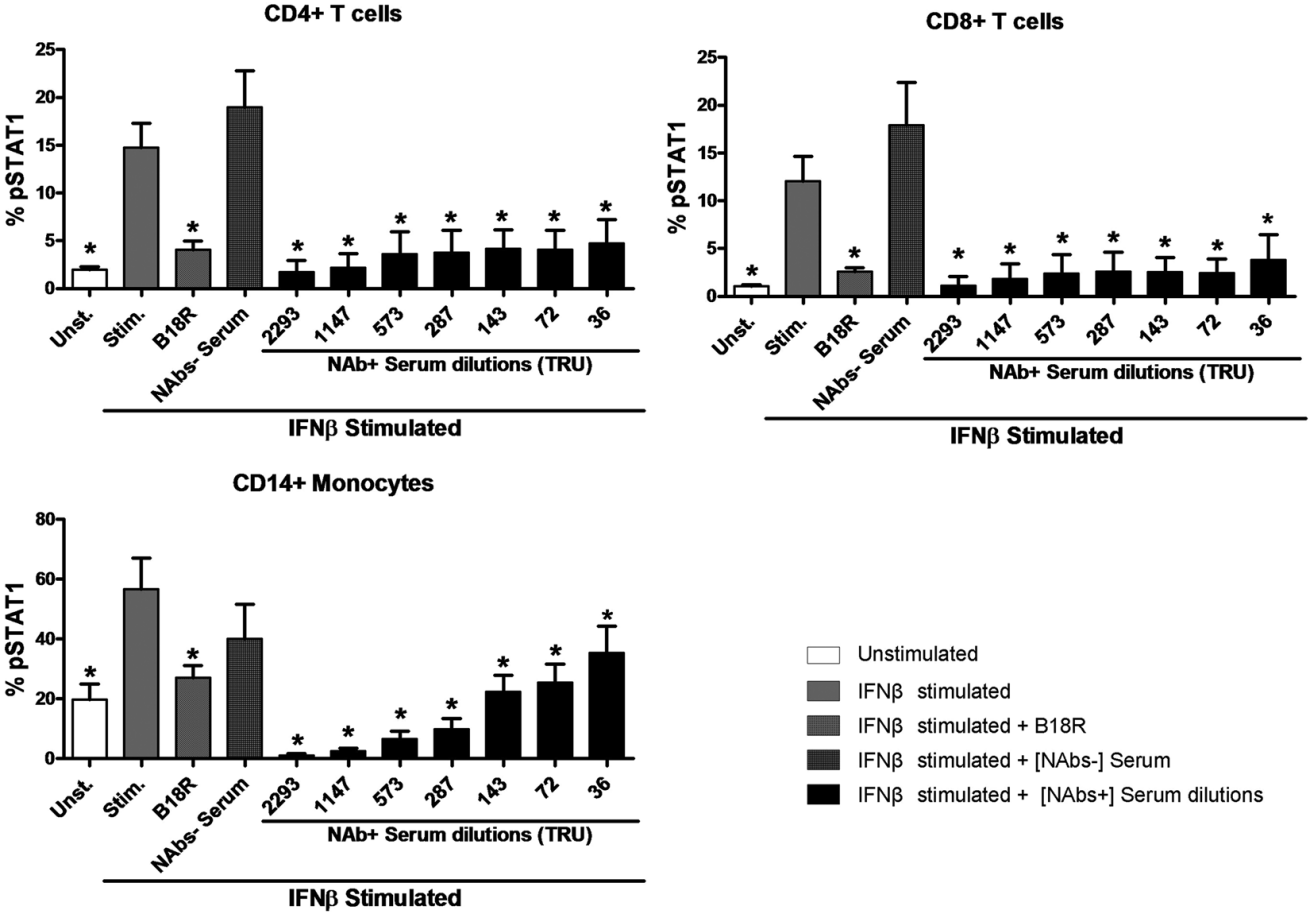
STAT1 phosphorylation measures according to the NAbs titers in PBMC from MS patients. Expression of pSTAT1 determined by phosphoflow in monocytes, CD4+ and CD8+ T lymphocytes from MS patients after stimulation with 1000 IU/mL IFNβ1a (Avonex) in presence of several dilutions of a NAbs positive heterologous serum. As controls, non stimulated cells, IFNβ stimulated cells with B18R inhibitor and IFNβ stimulated cells with a NAbs negative heterologous serum, were included. The activation of pSTAT1 achieved after IFNβ stimulation decreased significantly in presence of the different titers of NAbs compared to the activation without serum in T cells and monocytes. In presence of NAbs negative serum no decrease were detected. Data are shown as percentage of cells expressing pSTAT1. Significant p values are shown with asterisk: *p ≤ 0.05. *[NAb*+*]: positive neutralizing antibodies, [NAb*−*]: negative neutralizing antibodies*.

To evaluate the effect of the presence of NAbs in serum, the activation of STAT1 was compared to the activation achieved with only IFNβ. The STAT1 phosphorylation achieved in the presence of the different titers of NAbs was always significantly lower than that achieved in IFNβ stimulated cells, in CD4+, CD8+ T cells and monocytes. However, STAT1 phosphorylation showed no significant differences between IFNβ stimulated cells in the absence of serum and in the presence of a NAbs negative pool. Figure 2.

### Relationship between the STAT1 phosphorylation and NAbs titers

To analyze the association or interdependence between the STAT1 phosphorylation and NAbs titers, a correlation analysis in each independent patient was performed. A strong negative correlation was observed in all the patients, so that the STAT1 phosphorylation decreased as the NAbs titers increased. This association was statistically significant in the monocytes from all the patients and in most patients in the CD4+ and CD8+ T cells. Table 3.

**Table 3:** Correlations between pSTAT1 and NAbs titers in CD4+, CD8+ T cells and monocytes from MS patients.

	Correlations NAbs *vs* % of pSTAT1					
	CD4+ T cells		CD8+ T cells		Monocytes	
	r	p	r	p	r	p
Patient 1	−1.0000	0.0000	−0.9543	0.0008	−0.8929	0.0068
Patient 2	−0.9274	0.0026	−0.8982	0.0060	−0.9286	0.0025
Patient 3	−0.9643	0.0005	−1.0000	0.0000	−0.9636	0.0005
Patient 4	−0.7143	N.S.	−0.5357	N.S.	−1.0000	0.0000
Patient 5	−0.9274	0.0026	−0.8929	0.0068	−1.0000	0.0000
Patient 6	−0.8729	0.0103	−0.6108	N.S.	−0.8929	0.0068
Patient 7	−0.7748	0.0408	−0.8608	0.0129	−0.9643	0.0005
Patient 8	−0.5714	N.S.	−0.6307	N.S.	−0.8469	0.0162

r: Spearman Rho Correlation Coefficient; p: p values; *N.S*. non significant.

**Figure 3:**
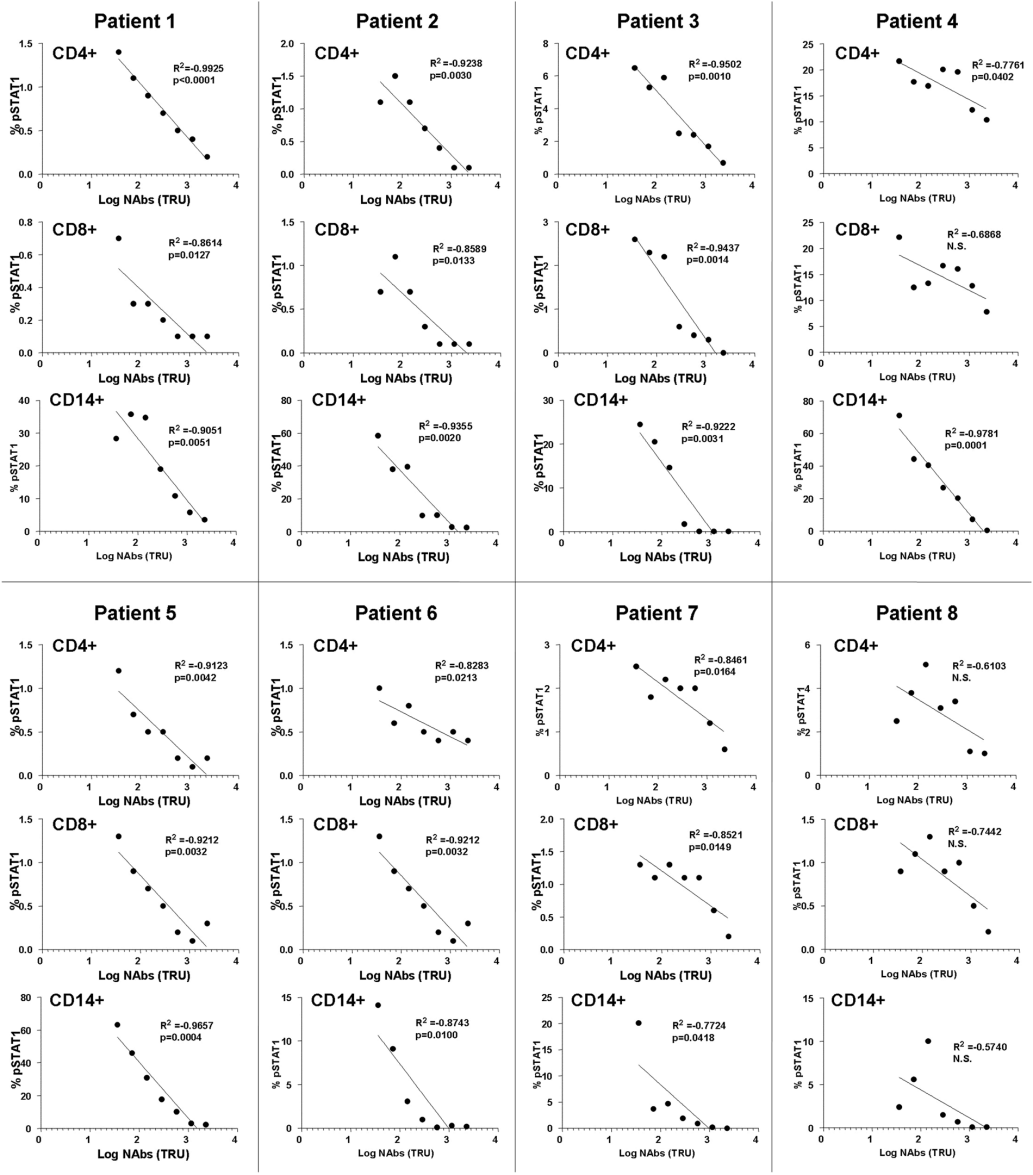
Lineal regression analysis between the percentage of pSTAT1 *versus* the logarithm of the NAbs titers in CD4+ and CD8+ T cells and in monocytes of each independent patient. R^2^: Coefficient of Determination (R Squared) p: p-value or probability value of the statistical model. N.S: non significant.

After that, the percentage of pSTAT1 *versus* the logarithm of the NAbs titers were represented showing a significant linear relationship between the two variables in T cells and monocytes from all the patients, except in patient 8 who did not reach significance for any of the cell subsets, nor CD8+ T cells in patient 4. Figure 3.

## Discussion

Although the NAbs quantification has been a controversial topic with relevant economic and clinical impact^11,12^, their detection is one of the most accepted biomarkers for monitoring response to IFNβ treatment^13^. High titers of NAbs can persist for years and their presence is associated with a loss of IFNβ bioactivity *in vivo* ^14^, which can translated into a loss of clinical efficacy of the drug^15^. There is a huge number of published papers focused on the effect of NAbs on the efficacy of treatment, however, in spite of the important consequences that cross-reactivity could have in the clinical practice, few of them have evaluated this fact^6–10^. In these studies cross-reactivity of NAbs with IFNβ-1a and IFNβ-b is described^6–9^ but not with IFNα-2a^10^.

Our research laboratory is a reference center for the monitoring of NAbs which offers this service to all the hospitals in Spain and Portugal. Consequently, we have an important collection including more than 1000 serum samples which have been tested for the presence of neutralizing antibodies, being each sample tested against three IFNβ preparations by CPE test according to the WHO recommendations^16^. From this historic data base, the cross-reactivity of NAbs against different IFNβ preparations and its association with NAbs titers has been evaluated.

Our data clearly shows that NABs are cross reactive with three different IFNß preparations and that the cross-reactivity occurs as higher the antibody titer is, so that patients with intermediate-high titers of antibodies have a 79-fold increased risk of cross-reactivity compared to patients with low titers. It is important to highlight that the estimation of this risk was done with a logistic regression model including 222 NAbs positives, being the grater cohort of patients used to evaluate the cross-reactivity against IFNβ.

Additionally to the evaluation of cross-reactivity by bioassay, which uses cell lines, it was also evaluated in PBMC from NAbs positives patients, by determining the activation of pSTAT1. As previously described by Gavasso *et al*., the presence of NAbs disrupt STAT activation in primary immune cell subtypes^17,18^. Our study not only corroborated that the presence of NAbs decreases the activation of pSTAT1 in reference to the activation achieved without NAbs but it also demonstrated the NAbs cross-reactivity, being these NAbs able to decrease the activation of pSTAT1 achieved by other different IFNβ molecules.

As far as we know, this is the first time in which an association between antibody titers and cross-reactivity is described, as well as the demonstration that NAbs inhibit the pSTAT1 activation achieved with other IFNβ molecules. So, these results strongly support the consensus guidelines on the clinical use of NAbs, in which it is not advised to switch from one type of IFNβ to another, nor increase the dose when NAbs are present^2^ because will probably be of no benefit.

In addition to the consequences of cross-reactivity on the efficacy of the administered drug, antibodies against therapeutic proteins are able to cross react and neutralize the native proteins. A clinical case in which an exacerbation of genital herpes coinciding with MS relapses has been described suggests that NAbs not only decreases exogenous IFNβ treatment efficacy, but may also interfere with anti-viral properties of endogenous IFNβ^19^. In addition, another study demonstrated that NAbs are able to interfere with endogenous type I interferons and that this interference could compromise immunoregulatory responses of MS patients^5^. The conclusions of this study, carried out in cell lines, are very relevant, but further studies are necessary to evaluate the consequences that the cross-reactivity of NAbs with endogenous IFNβ could have in MS patients.

The following objective of the study was to evaluate the inhibition of the STAT1 phosphorylation according to the NAbs titers. Previously, it was necessary to prove that, in the presence of an heterologous NAbs positive serum, a significant loss of STAT1 phosphorylation occurred, while, in the presence of an heterologous NAbs negative serum, the levels of pSTAT1 did not ever significantly decrease. Further experiments with serial dilutions of a pool of high positive heterologous sera were used to evaluate the response of pSTAT1 in the presence of low, intermediate and high levels of NAbs. Even low titers of NAbs were able to significantly decrease the activation of STAT1, abrogating the activation almost completely in the presence of high titers. The inhibitory effect on STAT1 activation observed with NAbs is supported with the inclusion of B18R, which is a decoy receptor with highly potent neutralizing activity of type I interferons^20^. Similarly as what occurs with this inhibitor, the phosphorylation achieved in the presence of low NAbs titers is very little compared with the phosphorylation obtained in IFNβ stimulated cells in the absence of serum and similar to that obtained in non stimulated cells. It is important to underline that, in monocytes, the presence of NAbs induced an even greater inhibition of pSTAT1 than the presence of B18R.

Previously, Gavasso *et al*.^18^ found that a deficient phosphorylation of STAT1 in leukocytes identified the presence of NAbs, in an *ex vivo* study using samples collected before and after IFNβ administration. In our approach, controlled *in vitro* stimulation conditions have been performed, including positive and negative controls, showing that the presence of NAbs clearly interfere in the JAk-STAT signaling pathway and that activation of STAT1 by phosphorylation is inversely correlated with the titers of NAbs. These results are completely in concordance with those from Gavasso *et al*. but, additionally demonstrate that a linear relationship between levels of pSTAT1 and NAbs titers exists. According to the European guidelines, NAbs should be quantified in each patient after one year of IFNβ therapy and, in case of persistent NAb-positivity with high titers, IFNβ should be discontinued^21^. However, our experiments highlight the importance of low NAbs in clinical practice. Thus, it seems reasonable to perform measurements of NAbs in all the IFNβ-treated patients to identify those positive for NAbs and to avoid the risk of some patients of being treated for a long time with limited efficacy.

Coming back to the relationship between the levels of pSTAT1 and the NAbs titers, the analysis of each independent patient showed a strong negative correlation, so that the STAT1 phosphorylation decreased as the NAbs titers increased. A linear relationship between the percentage of pSTAT1 and the logarithm of the NAbs titers was observed, in all but one patient. Although the linear relationship was observed in the three cellular subsets analyzed, it is in monocytes where the loss of STAT1 activation in the presence of NAbs is better reflected.

Definitely, the bioassay continues being the gold standard for determining neutralizing antibodies, but, our approach clearly suggest that pSTAT1 determination by flow cytometry in monocytes could be an alternative quicker method for the detection of NAbs, being necessary further analytical validation for its implementation in clinical practice.

In conclusion, our study demonstrates that patients with intermediate and high titers of NAb had higher risk of cross reactivity with the different exogenous IFNβ molecules therapeutically administered, being of particular interest at the time to switch or increase the dose of IFNß treatment. But also low titers should be considered, since they are able to decrease the levels of pSTAT1. In that way, the activation of STAT as a key step for the IFNβ signaling, is dependent of the titer of NAbs and its determination by flow cytometry could be an indirect method to identify the presence of NAbs.

## Material and Methods

### Subjects

#### NAbs Cross-reactivity study

The research laboratory of the Hospital Civil in Málaga (IBIMA) is a reference centre in Spain and Portugal for the analysis of neutralizing antibodies (NAbs) against IFNβ, performed ad hoc at the request of the neurologists. Therefore, a collection of more than 1000 sera from MS patients treated with IFNβ is available. In each of these samples, the presence of antibodies against three of the four commercially IFNβ molecules (Avonex™, Rebif™ y Betaferon™) has been determined by CPE test, regardless of the molecule received as treatment.

For the cross-reactivity study, from this historic database, 222 NAbs positive patients, defined by a titer ≥20 TRU/ml against one of the three IFNβ molecule tested, were selected.

Patients signed informed consent in their respective hospitals for the analysis of neutralizing antibodies. In addition, all methods of this work were performed in accordance with the relevant guidelines and regulations after approval by institutional ethical committees of our hospital (Comité de Ética de la Investigación provincial de Málaga). Each of the requesting neurologists has been informed of the completion of the study and the dissemination of the results.

#### Interference of the presence of NAbs in the activation of the IFNβ signaling pathway

Ten NAbs positive patients treated with Rebif were selected to evaluate how an autologous serum with NAbs interferes in the STAT1 phosphorylation and the cross-reactivity. In addition, eight NAbs negative patients were confronted with a heterologous NAbs positive serum to evaluate pSTAT1 according to the NAbs titers. The demographic and clinical characteristics of patients included in theses experiments are shown in Table 4.

**Table 4:** Demographic and clinical characteristics of the MS patients included in pSTAT1 experiments.

Female/male	14/4
Age (years)	39,7 ( ± 13.7)
MS duration (years)	11.7 ( ± 14.5)
EDSS	1.5 ( ± 1.1)
IFNß treatment (Avonex/Betaferon/Rebif)	(1/3/14)
NAbs titter (high/intermediate/low)	(6/1/3)

MS: multiple sclerosis; EDSS: Expanded Disability Status Scale.

Age, MS duration and EDSS are presented as mean ± standard deviation.

The samples were provided by the Biobank of our hospital, as part of the Andalusian Public Health System Biobank. All patients participating in the study gave their informed consent and protocols were approved by institutional ethical committees (Comite de Ética de la Investigación provincial de Málaga).

### Sample collection

Peripheral blood was drawn by venipuncture from MS patients. The sera were decomplemented at 56 °C for 30 min and were cryopreserved until their use. Peripheral blood mononuclear cells (PBMC) were isolated using a ficoll-hypaque gradient, as described in the supplier’s protocol (ICN Biomedicals Inc., OH, USA). After that, cells were cryopreserved in RPMI-1640 medium supplemented with 40% heat-inactivated fetal calf serum (FCS) and 10% DMSO, until use.

### NAbs determination by CPE test

Sera of IFNβ-treated patients were analysed for the presence of neutralizing antibodies against three commercial forms of IFNβ (IFNβ1a (Avonex, Biogen, Inc.), IFNβ1b (Betaferon, Bayer Pharma AG) or IFNβ1a (Rebif, Merck Serono Ltd) by a calibrated antiviral cytopathic effect assay, as previously described by Oliver *et al*.^16^. Briefly, A549 cells were seeded in 96 well plates in 100 μl of DMEM medium supplemented with 2% FCS. After 24 h, serial dilutions of the patient sera (1/10–1/640) were incubated for 1 h with commercially IFNβ, and the same dilutions without IFNβ were placed in another column to measure the presence of endogenous IFNβ in the patient serum. These dilutions were added to the A549 cell culture, sown the day before. Each plate included a viral control which did not contain IFNβ, a cell control which did not contain virus, and a standard of IFNβ (serial dilutions: 1/1 to 1/128). After 24 h incubation, cells were infected with Encephalomyocarditis virus (EMCV) (except for the cell control, to which only DMEM medium without serum was added). Twenty four hours later, the cells were stained with crystal violet and the absorbance was read at 630 lambdas in a spectrophotometer. The neutralization titre of a serum sample was calculated according to Kawade *et al*.^22^ and expressed in 10-fold reduction units per millilitre (TRU/mL). Titres ≥20 TRU/mL were considered as positive.

### Categories of NAbs titers and definition of cross-reactivity

The antibody titers was categorized as follows: low (≤100 TRU/ml), intermediate (>100 and ≤300 TRU/ml) and high (>300 TRU/mL). This classification was made based on our experience and that of others^17,23^.

By definition in immunology, the cross-reactivity is the reaction between an antibody and an antigen that differs from the immunogen. It was considered that a patient had cross-reactivity when the antibodies generated against the IFNβ molecule received as treatment were able to react, at least, to one of the two other IFNβ molecules analyzed.

### Cell cultures and determination of pSTAT1 by flow cytometry

PBMC from MS patients were thawed and suspended in prewarmed RPMI-1640 medium supplemented with 2 mM l-glutamine, 20% FCS and 0.032 mg/ml gentamicin. Cells were washed by centrifugation, resuspended in the same medium without FCS (1 million of cells/ml) and incubated at 37 °C for 90 min, in order to obtain the lowest level of activation.

After that, the cells were stimulated as follows:

1. To evaluate the interference of the presence of NAbs in the activation of the IFNβ signaling pathway, as well as the cross-reactivity, the cells of each NAbs positive patient were exposed to the following stimulation conditions during 30 minutes at 37 °C to allow the signal transduction and the STAT1 phosphorylation:

- 1000 UI/mL of IFNβ (Rebif, Avonex or Betaferon), considered as reference.
- 1000 UI/mL of IFNβ (Rebif, Avonex or Betaferon) + autologous NAbs positive serum (20%).
- 1000 UI/mL of IFNβ (Rebif, Avonex or Betaferon) + soluble B18R (Millipore), a decoy receptor for Type I interferons which has potent neutralizing activity.

2. To evaluate the inhibition of STAT1 phosphorylation according to the NAbs titers, the cells from 10 patients were exposed during 30 minutes at 37 °C to IFNβ stimulation (1000 UI/mL of Avonex) to allow the STAT1 phosphorylation in the presence of serial dilutions of a heterologous pool of positive NAbs sera. For that purpose, this pool sera was titered by bioassay (4586 TRU) and was diluted to 1/2, 1/4, 1/8, 1/16, 1/32 1/64 to obtained high (2293, 1147 and 573 TRU), intermediate (287 and 143 TRU) and low titter (72 and 36 TRU) of NAbs. IFNβ stimulated cells were considered as reference. The following controls were included: a. unstimulated cells. b. IFNβ stimulated cells with B18R inhibitor. c. IFNβ stimulated cells in presence of a pool of heterologous NAbs-negative sera.

Following stimulation in each experiment, the cells were fixed with Cytofix at 37 °C for 10 min, washed twice with Perm/Wash Buffer and permeabilized with PermBuffer III (all from BD Biosciences) at 4 °C for 20 min. After two additional washes, cells were stained for 30 min with, phycoerythrin-cyanine, peridin chlorophyll protein, Alexa Fluor-488 and allophycocyanin labelled specific monoclonal antibodies (MAB) for the following molecules: phospho-STAT1 (Y^701^), CD3, CD8 and CD14 (all from BD Biosciences). This four colour staining with the MoAb were performed in order to evaluate the activation of STAT1 in the different cell populations. Isotype-matched controls were used to verify the staining specificity of the antibodies.

**Figure 4:**
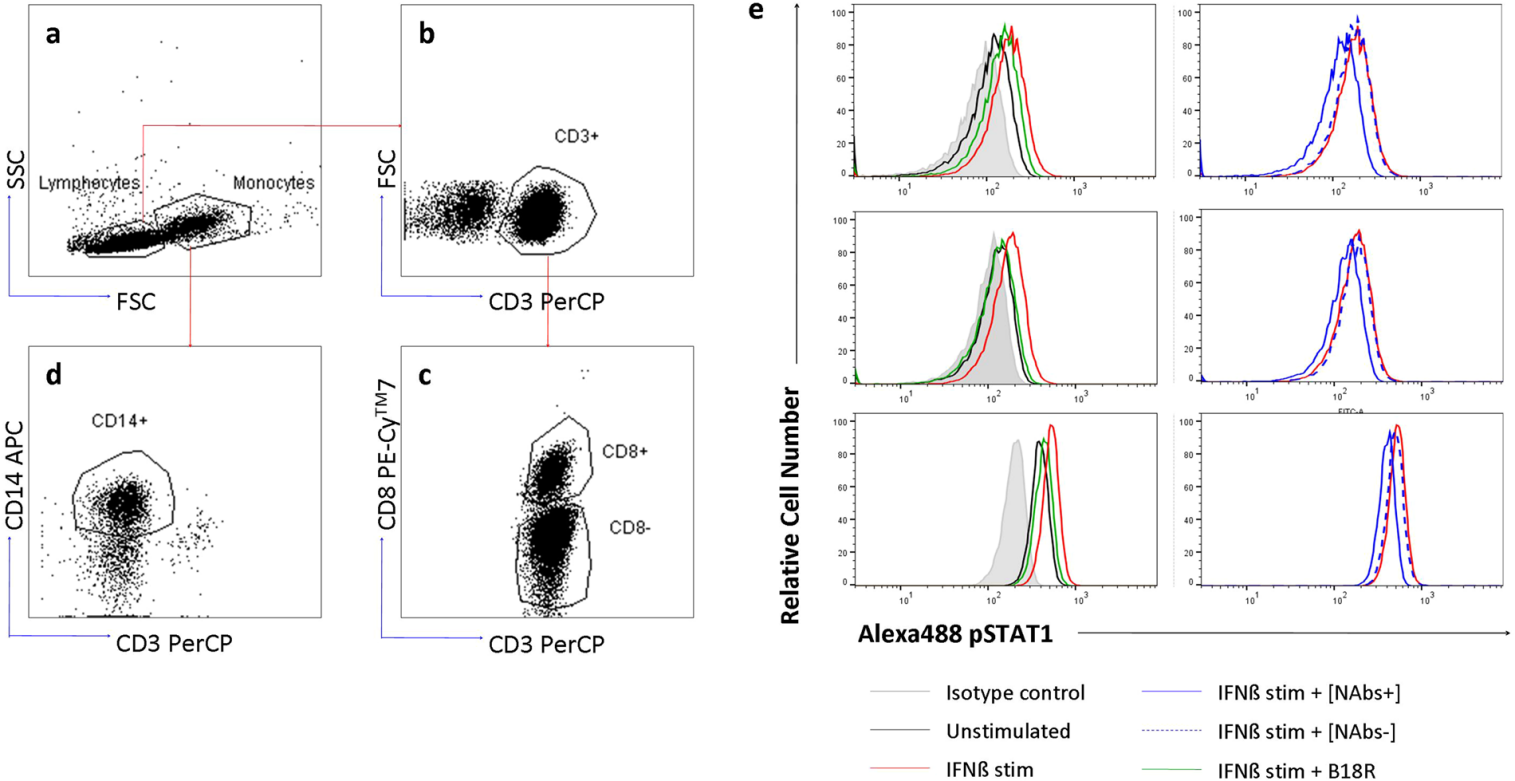
Gating strategy to determine pSTAT1 expression on different PBMC subsets. Based on the combination of side (SSC) and forward (FSC) light scatter properties of the acquired events, lymphocyte and monocyte gates were selected (**a**). T lymphocytes were identified by gating on CD3+ cells (**b**), then, they were transferred to a new dot plot and were analyzed by a specific antibody against CD8. T CD8+ lymphocytes were identified as CD3+ and CD8+ cells and T CD4+ lymphocytes as CD3+ and CD8- cells (**c**). In a new dot, monocytes were identified as CD14+ and CD3- cells (**d**). Overlay histograms from a representative patient that depict CD4+, CD8+ T lymphocytes and monocytes expressing pSTAT1 in the different cells stimulation conditions: unstimulated cells (black line), IFNβ stimulated cells (red line), IFNβ stimulated cells in presence of B18R inhibitor (green line), IFNβ stimulated cells in presence of NAbs positive serum (blue line), IFNβ stimulated cells in presence of NAbs negative serum (blue dashed line) and isotype control (shaded histograms) (**e**). *PBMC: peripheral blood mononuclear cells*, *FSC: forward* scatter, *SSC: side scatter, PE-Cy7: phycoerythrin-cyanine, PerCP: peridin chlorophyll protein, Alexa488: Alexa Fluor-488, APC: allophycocyanin*.

At least 50,000 events from each sample were acquired in a FACSCanto II™ flow cytometer using the FACSDiva software (BD Biosciences). Monocytes and lymphocytes were first gated based on side and forward light scatter properties. Monocytes were identified as CD3^−^CD14^+^ cells and, CD4^+^ lymphocytes were detected as CD3^+^ CD8^−^ cells, and CD8^+^ lymphocytes were distinguished as CD3^+^ CD8^+^ cells. The expression of pSTAT1 was determined in CD4^+^, CD8^+^ T cells and monocytes, for the different stimulation conditions. The data were analyzed as mean fluorescence intensities (MFI) and percentage of cells expressing pSTAT1 (% pSTAT1^+^). The gating strategy and a representative example of the histograms of STAT1 obtained in the different cells stimulation conditions is showed in Fig. 4.

### Statistical analysis

The relationship between NAbs titers and cross-reactivity was analyzed using the chi-square test. Subsequently, a logistic regression analysis was performed to calculate the risk of cross reactivity according to the antibody titers and after, a ROC (Receiver Operating Characteristic) curve analysis was also performed.

For the *in vitro* studies, Wilcoxon Rank test (related samples analysis) was used to compare the MFI and % pSTAT1+ in IFNβ-stimulated cells in presence of NAbs compared to IFNβ-stimulated cells without NAbs.

To analyze the association or interdependence between the STAT1 phosphorylation and the NAbs titers, Spearman’s Rho correlation analysis was used, followed by a lineal regression analysis.

Figures have been created with GraphPad Prism5.

## Electronic supplementary material

Supplementary Information
