# Supplementary material for: Cross-reactivity of antibodies against interferon beta in multiple sclerosis patients and interference of the JAK-STAT signaling pathway
Source: Sci Rep. 2017 Nov 29;7:16585. doi: 10.1038/s41598-017-16828-x (PMC5707350; doi:10.1038/s41598-017-16828-x)
Supplement: Supplementary file 1 — Supplementary Information [file 41598_2017_16828_MOESM1_ESM.pdf]

TITLE: Cross-reactivity of antibodies against interferon beta in multiple sclerosis patients and interference of the JAK-STAT signaling pathway.

Authors: Isaac Hurtado-Guerrero PhD, Maria Jesus Pinto-Medel PhD, Patricia Urbaneja, MD, Jose Luis Rodriguez Bada, Jesús Ortega-Pinazo, Pedro Serrano MD PhD, Óscar Fernández MD PhD, Laura Leyva, MD PhD, Begoña Oliver-Martos, PhD

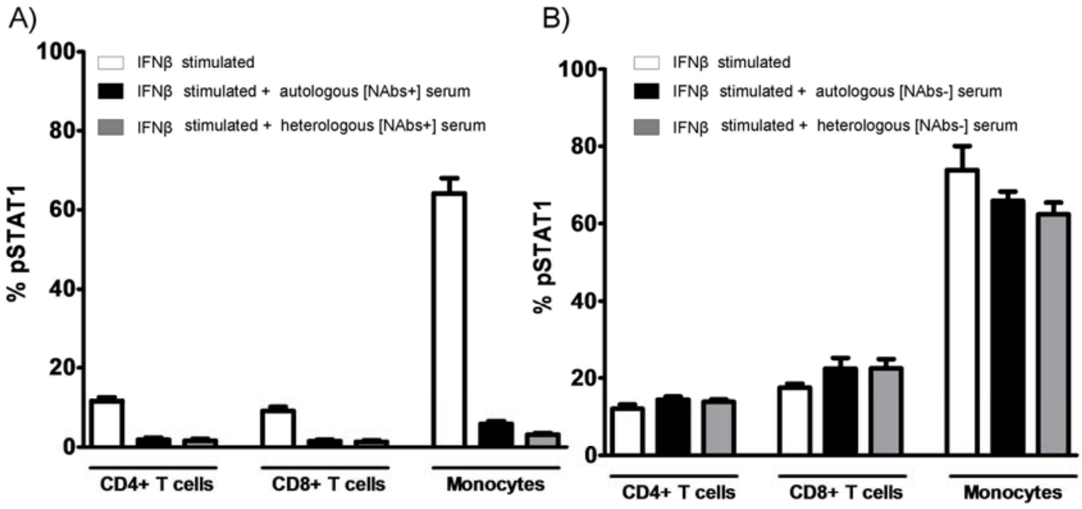

Supplementary figure 1

A) Expression of pSTAT1 determined by phosphoflow in CD4 + and CD8 + T lymphocytes and monocytes from MS patients after stimulation with 1000 IU/mL IFNβ1a (Avonex) and in presence of a NAbs positive autologous and heterologous serum. B) The same experiment was performed in presence of a NAbs negative autologous and heterologous serum. The graphics show that the decrease of pSTAT1 occurred in presence of an autologous NAbs positive sera but also in presence of an hererologous NAb positive sera. Autologous and hererologous NAb negative sera not decreased the level of pSTAT1.
